# Application of beta and gamma carbonic anhydrase sequences as tools for identification of bacterial contamination in the whole genome sequence of inbred Wuzhishan minipig (Sus scrofa) annotated in databases

**DOI:** 10.1093/database/baab029

**Published:** 2021-05-18

**Authors:** Reza Zolfaghari Emameh, Seyed Nezamedin Hosseini, Seppo Parkkila

**Affiliations:** Department of Energy and Environmental Biotechnology, National Institute of Genetic Engineering and Biotechnology (NIGEB), 14965/161, Tehran, Iran; Department of Recombinant Hepatitis B Vaccine, Production and Research Complex, Pasteur Institute of Iran, Tehran, Iran; Faculty of Medicine and Health Technology, Tampere University, Tampere, Finland; Fimlab Ltd, Tampere University Hospital, Tampere, Finland

## Abstract

*Sus scrofa* or pig was domesticated thousands of years ago. Through various indigenous breeds, different phenotypes were produced such as Chinese inbred miniature minipig or Wuzhishan pig (WZSP), which is broadly used in the life and medical sciences. The whole genome of WZSP was sequenced in 2012. Through a bioinformatics study of pig carbonic anhydrase (CA) sequences, we detected some β- and γ-class CAs among the WZSP CAs annotated in databases, while β- or γ-CAs had not previously been described in vertebrates. This finding urged us to analyze the quality of whole genome sequence of WZSP for the possible bacterial contamination. In this study, we used bioinformatics methods and web tools such as UniProt, European Bioinformatics Institute, National Center for Biotechnology Information, Ensembl Genome Browser, Ensembl Bacteria, RSCB PDB and *Pseudomonas* Genome Database. Our analysis defined that pig has 12 classical α-CAs and 3 CA-related proteins. Meanwhile, it was approved that the detected CAs in WZSP are categorized in the β- and γ-CA families, which belong to *Pseudomonas* spp. and *Acinetobacter* spp. The protein structure study revealed that the identified β-CA sequence from WZSP belongs to *Pseudomonas aeruginosa* with PDB ID: 5JJ8, and the identified γ-CA sequence from WZSP belongs to *P. aeruginosa* with PDB ID: 3PMO. Bioinformatics and computational methods accompanied with bacterial-specific markers, such as 16S rRNA and β- and γ-class CA sequences, can be used to identify bacterial contamination in mammalian DNA samples.

## Introduction

Pigs (*Sus scrofa*) were domesticated in multiple geographic regions of Asia and Europe through artificial and natural selections about 10 000 years ago. Especially in China as one of the main centers, the domestication created a number of indigenous breeds with various phenotypes including Plateau, Lower Yangtze River Basin, Southwest and North China types (1–3). The whole genome sequences (WGS) of pig models and minipig varieties are important in biomedical studies, such as generation of porcine-induced pluripotent stem cells for the treatment of human diseases including diabetes and cancer as well as ophthalmic, neurodegenerative and cardiovascular diseases (4, 5).

Wuzhishan pig (WZSP) is a Chinese inbred miniature minipig, which is characterized by its small size, approximately weight of 30 kg, homozygosis, genetic stability and good predictability in *in vivo* studies (6). WZSP was developed in the Institute of Animal Science of the Chinese Academy of Agriculture Science in 1987. Fang *et al.* performed the WGS of WZSP in 2012, which defined a high-level derivation of transposons from transfer RNA with 2.2 million copies (12.4% of the genome) (7). In addition, many human gene and effective drug targets have been identified in the genome of WZSP. The WGS of WZSP, completed by the researchers from Beijing Genomics Institute, provided pivotal data for the use of this minipig model in biological, medical and veterinary medicine studies.

The genome of WZSP contains porcine endogenous retroviruses (PERVs), which can be transmitted in the germ lines and infect human cells, leading to severe combined immunodeficiency (8). Therefore, PERVs are considered a great potential risk of xenotransplantation of organs from transgenic pigs like WZSP to human.

Carbonic anhydrases (CAs) are ubiquitous enzymes with metal cofactors such as zinc, iron, cobalt or cadmium in the enzyme active sites catalyzing the hydration of CO_2_ to HCO_3_^−^ and H^+^ for pH homeostasis and playing the crucial roles in many biochemical pathways and physiological functions (9, 10). CAs are classified into eight evolutionarily distinct families, including α, β, γ, δ, ζ, η, θ and ι (11–14). α-CAs are present in many prokaryotes and eukaryotes (15, 16). There are 13 α-CA isozymes in mammals, of which 12 are present in humans, including CA I–IV, CA VA and VB, CA VI, CA VII, CA IX and CA XII–XIV. CA XV can be found in several vertebrates with the exception of at least chimpanzee and human (17). In addition, the presence of three acatalytic CA-related proteins (CARPs), including CARP VIII, CARP X and CARP XI, has been reported, and these highly conserved proteins seem to play critical biological roles (18–22). Although β- and γ-CAs have been reported in several prokaryotes and eukaryotes, there is no report showing the presence of a β- or γ-CA in vertebrates (23, 24).

Databases such as Ensembl Genome Browser contain huge data resources of vertebrate genomes to support the related studies in various fields, such as evolutionary and computational biology, associated with the WGS, gene expression studies and encoded protein analyses in vertebrates (25). Due to the bacterial contamination of eukaryotic nucleic acid samples with environmental microbiome and normal flora of the eukaryotic hosts, some contaminant gene and protein sequences from prokaryotes have been erroneously annotated for eukaryotes in databases (26).

In this study, we performed a quality control analysis of the WGS results of WZSP annotated in databases using *β-* and *γ-CA* gene sequences as markers through bioinformatics and data mining approaches.

## Methods

### Identification of CAs from *S. scrofa*

To identify genomics and proteomics information of the CA isozymes from *S. scrofa*, the National Center for Biotechnology Information (NCBI) database (https://www.ncbi.nlm.nih.gov/) (27) was used to define the chromosome location and exon counts of the corresponding genes. In addition, data from the UniProt database (https://www.uniprot.org/) (28) were used to define the subcellular localization of CA isozymes from *S. scrofa.*

### Analysis of β- and γ-CA sequences

In this analysis, β-CA protein sequence from *Acetobacter aceti* (UniProt ID: A0A1U9KGA1) and γ-CA protein sequence from *Shigella flexneri* (UniProt ID: P0A9X0) were used as the query sequences. Basic Local Alignment Search Tool (BLAST) analysis was performed on both β- and γ-CA query sequences using BLAST algorithm of Ensembl Genome Browser (https://asia.ensembl.org/index.html) (25). To find similar sequences in the BLAST analysis, Pig-Wuzhishan (assembly: minipig_v1.0; accession: GCA_002844635.1; genebuild released: September 2019) was selected by species selector section, and TBLASTN search tool with normal sensitivity was applied to search for the translated nucleotide databases using a protein query. In the next step, the defined β- and γ-CA protein sequences of WZSP were analyzed by the BLAST homology search tool of the UniProt database. In the final step, multiple sequence alignment (MSA) analysis was performed on all β- and γ-CA protein sequences involved in this evaluation using Clustal Omega algorithm of the European Bioinformatics Institute database (https://www.ebi.ac.uk/Tools/msa/clustalo/) (29). To reduce the size of protein sequences and output figures from MSA analysis, just 69 and 60 amino acid sequences of β- and γ-CA protein sequences containing the enzyme active sites were selected, respectively.

### Genomic analysis of β- and γ-CA sequences from putative bacterial contaminants

The coding genes for β- and γ-CAs from *Pseudomonas* spp. as one of the putative contaminants in WGS of WZSP were evaluated using the BLASTP search tool in the *Pseudomonas* Genome Database, version 20.2 (https://www.pseudomonas.com/) (30) by using 1e-4 as the default value cutoff. In addition, the coding genes for β- and γ-CAs from *Acinetobacter* spp. as another potential contaminant were analyzed by the Ensembl Bacteria database (http://bacteria.ensembl.org/index.html) (31).

### Protein structure analysis

Four β-CA protein sequences from bacterial contaminates including UniProt IDs: A0A0Q8Y2C1, A0A4R3W4C9, A0A656JXK1 and A0A062C2I7 and six γ-CA protein sequences from bacterial contaminants including UniProt IDs: A0A4R3W1J2, A0A125QD08, A0A4R3W9L6, A0A2N1E8I6, A0A062BNN8 and A0A419V156 were analyzed by RCSB Protein Data Bank (PDB) (https://www.rcsb.org/) (32) to identify the most similar crystallized and 3D model proteins to the query β- and γ-CA protein sequences of bacterial contaminants.

## Results

### Identification of α-CAs from *S. scrofa*

This analysis defined 12 α-CA isozymes including CA I–IV, CA VA and VB, CA VI, CA VII, CA IX and CA XII–XIV and three CARPs including CARP VIII, CARP X and CARP XI in *S. scrofa*. The results revealed that chromosome 1 contains the coding genes for CA IX and CA XII; chromosome 4 contains the coding genes for CA I–III, CA XIII, CAXIV and CARP VIII; chromosome 6 contains the coding genes for CA VA, CA VI, CA VII and CARP XI; chromosome 12 contains the coding genes for CA IV and CARP X and chromosome X contains the coding gene for CA VB. Our study on the subcellular localization of α-CAs from *S. scrofa* predicted that CA I–III, CA VII, CA XIII and CARP VIII are cytoplasmic; CA VA and CA VB are mitochondrial; CA VI, CARP X and CARP XI are secretory; CA IX, CA XII, and CA XIV are transmembrane and CA IV is membrane-bound (Table 1).

**Table 1.** α-CAs from *S. scrofa*

α-CAs	UniProt ID	NCBI ID	Gene location	Exon count	Subcellular localization
CA I	A0A287AI92	XP_001924218.1	Chromosome 4	7	Cytoplasmic
CA II	A0A287B6M0	XP_001927840.1	Chromosome 4	7	Cytoplasmic
CA III	A0A4X1UEH4	NP_001008688.1	Chromosome 4	7	Cytoplasmic
CA IV	F1S1C3	NP_001230849.1	Chromosome 12	8	Membrane-bound
CA VA	A0A5G2QRM5	XP_020949335.1	Chromosome 6	13	Mitochondrial
CA VB	F1SQS9	XP_005673507.1	Chromosome X	9	Mitochondrial
CA VI	F1RIH8	NP_001137588.1	Chromosome 6	8	Secretory
CA VII	A0A286ZZG4	XP_020949678.1	Chromosome 6	8	Cytoplasmic
CA IX	A0A5G2QGY0	XP_001925555.2	Chromosome 1	12	Transmembrane
CA XII	F1S092	XP_020949824.1	Chromosome 1	11	Transmembrane
CA XIII	A0A287ASJ5	XP_001924497.3	Chromosome 4	9	Cytoplasmicc
CA XIV	A0A287B0I5	XP_020945576.1	Chromosome 4	9	Transmembrane
CARP VIII	A0A287BFY8	XP_020944998.1	Chromosome 4	10	Cytoplasmic
CARP X	A0A480LJN7	XP_020922898.1	Chromosome 12	11	Secretory
CARP XI	A0A4X1VZX6	XP_005664726.1	Chromosome 6	9	Secretory

### Analysis of β- and γ-CA sequences

**Figure 1.**
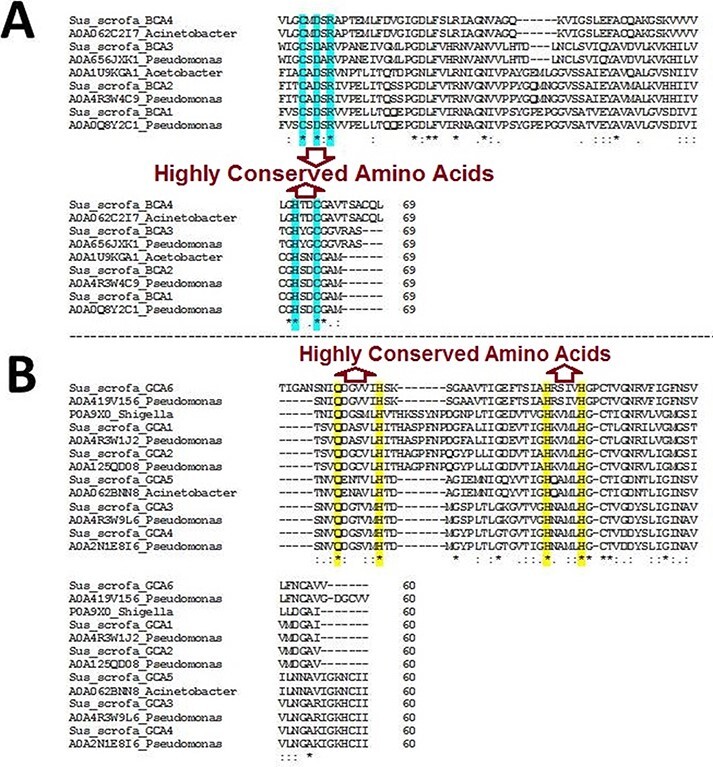
Multiple sequence alignment (MSA) of β- and γ-CA sequences. (A) MSA of β-CA sequences shows highly conserved amino acids in cyan color; (B) MSA of γ-CA sequences shows highly conserved amino acids in yellow color.

The BLAST homology analysis of the predicted WZSP CA sequences first identified a β-CA sequence from *A. aceti* and a γ-CA sequence from *S. flexneri*. A more detailed BLAST homology analysis of β-CA and γ-CA sequences from WZSP showed 100% similarity with bacterial β- and γ-CA sequences from *Pseudomonas* spp. and *Acinetobacter* spp. To confirm the identity of the defined sequences, MSA of the β-CA sequences showed the five highly conserved amino acids, including cysteine, aspartic acid, arginine (CXDXR) and histidine and cysteine (HXXC), which are known to be characteristic features of β-CA enzymes. Similarly, the predicted γ-CA sequences showed the four highly conserved amino acids characteristic of γ-CAs, including glutamine and histidine (QXXXXXH) as well as two histidines (HXXXXH) (Table 2; Figure 1).

**Table 2.** List of β- and γ-CA sequences from WZSP with 100% identity to counterpart sequences from bacteria

				TBLASTN results					
CA family	CA query (UniProt ID)	WZSP CA (Ensmbl genomic location)		Length (amino acids)	ID (%)	Bacteria (UniProt ID)	*E*-value	ID (%)	RSCB PDB 3D model
β-CA	*Acetobacter aceti* (A0A1U9KGA1)	BCA1	AJKK01119664: 532–1149	222	46.40	*Pseudomonas* sp. (A0A0Q8Y2C1)	7e-59	100	5JJ8
		BCA2	KQ002894: 52 809–53 450	203	52.22	*Pseudomonas* sp. (A0A4R3W4C9)	2e-64	100	
		BCA3	AJKK01121845: 27–380	109	36.70	*Pseudomonas syringae* (A0A656JXK1)	5e-12	100	
		BCA4	AJKK01117230: 2023–2607	176	26.14	*Acinetobacter* sp. (A0A062C2I7)	1e-09	100	
γ-CA	*Shigella flexneri* (P0A9X0)	GCA1	KQ002894: 61 481–62 005	175	60.57	*Pseudomonas* sp. (A0A4R3W1J2)	6e-71	100	3PMO
		GCA2	AJKK01118454: 663–1190	176	61.36	*Pseudomonas fluorescens* (A0A125QD08)	1e-71	100	
		GCA3	KQ002836: 4671–5114	155	38.06	*Pseudomonas* sp. (A0A4R3W9L6)	2e-28	100	
		GCA4	AJKK01180312: 124–558	152	37.50	*Pseudomonas fluorescens* (A0A2N1E8I6)	9e-27	100	
		GCA5	AJKK01118286: 1328–1756	150	35.33	*Acinetobacter* sp. (A0A062BNN8)	3e-25	100	
		GCA6	AJKK01161219: 1382–1714	119	34.45	*Pseudomonas synxantha* (A0A419V156)	2e-13	100	

### Genomic analysis of β- and γ-CA sequences from putative bacterial contaminants

**Figure 2.**
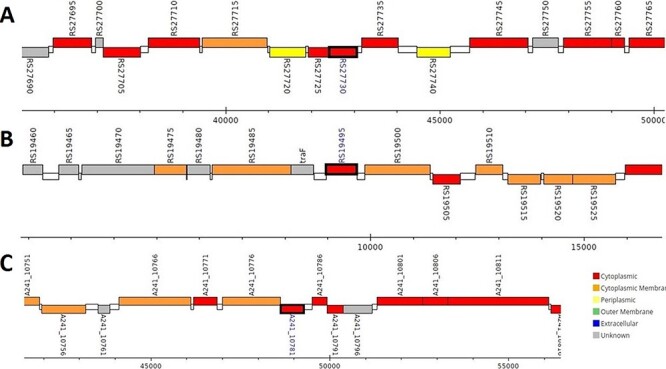
Genomic analysis of β-CA sequences from putative contaminants associated with *Pseudomonas* spp. The analysis shows the presence of coding genes for β-CA from (A) *Pseudomonas* sp. (UniProt ID: A0A0Q8Y2C1), (B) *Pseudomonas* sp. LP_8_YM (UniProt ID: A0A4R3W4C9) and (C) *Pseudomonas syringae* pv. actinidiae ICMP 19096 (UniProt ID: A0A656JXK1).

**Figure 3.**
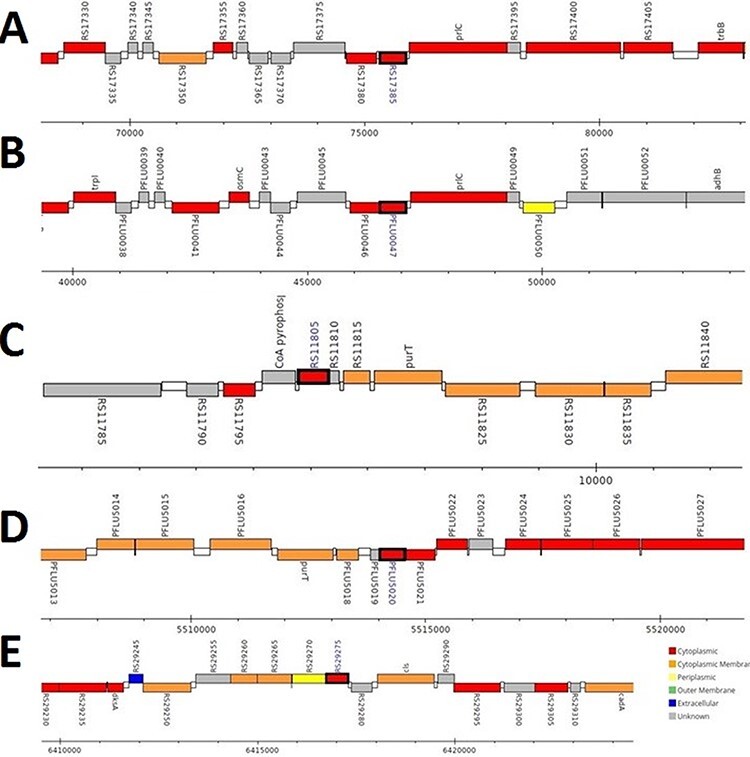
Genomic analysis of γ-CA sequences from putative contaminants associated with *Pseudomonas* spp. The analysis shows the presence of coding genes for γ-CA from (A) *Pseudomonas* sp. LP_8_YM (UniProt ID: A0A4R3W1J2), (B) *Pseudomonas fluorescens* (UniProt ID: A0A125QD08), (C) *Pseudomonas* sp. LP_8_YM (UniProt ID: A0A4R3W9L6), (D) *Pseudomonas fluorescens* (UniProt ID: A0A2N1E8I6) and (E) *Pseudomonas synxantha* (UniProt ID: A0A419V156).

**Figure 4.**
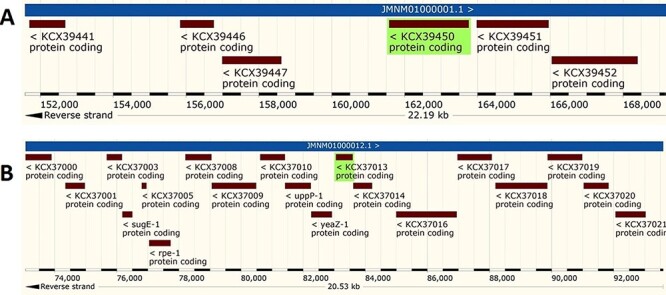
Genomic analysis of β- and γ-CA sequences from putative contaminants associated with *Acinetobacter* spp. The analysis shows the presence of coding genes for (A) β-CA from *Acinetobacter* sp. 263903-1 (UniProt ID: A0A062C2I7) and (B) γ-CA from *Acinetobacter* sp. 263903-1 (UniProt ID: A0A062BNN8).

The analysis revealed that the *β-* and *γ-CA* genes from putative bacterial contaminants are located in the genomes of *Pseudomonas* spp. and *Acinetobacter* spp. Further evaluation revealed that all the encoded β- and γ-CAs from the putative bacterial contaminants are probably cytoplasmic proteins (Figures 2–4).

### Protein structure analysis

**Figure 5.**
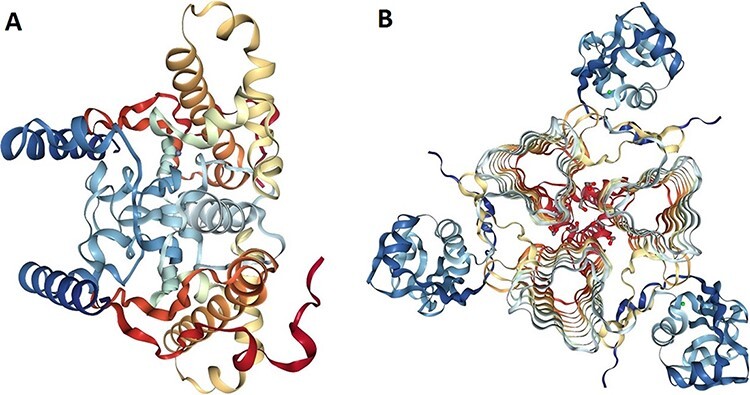
Protein structure analysis of β- and γ-CA protein sequences from bacterial contaminants. (A) Accession ID: 5JJ8 crystal structure belongs to β-CA from *P. aeruginosa*, and (B) Accession ID: 3PMO crystal structure belongs to γ-CA from *P. aeruginosa*. A and B were obtained from the PDB database, which are the most similar crystalized structures to β- and γ-CAs from bacterial contaminants, respectively.

The 3D models of crystallized β- and γ-CA protein structures, most similar to the bacterial contaminant proteins described in this study, were visualized in NGL (WebGL) viewer of the RSCB PDB database (accession codes 5JJ8 and 3PMO) (Figure 5). The visualized images of the bacterial β- and γ-CA proteins show homodimeric and homotrimeric structures typical for the β- and γ-CA proteins, respectively (33).

## Discussion

α-CAs have been classically considered the only CA family that is present in vertebrates. In line with those observations, our study revealed that *S. scrofa* has 12 α-CA isozymes and 3 CARPs similar to human (26). These α-CAs have subcellular localizations that are concordant with human enzymes, including cytoplasmic CA I–III, CA VII, CARP VIII and CA XIII; membrane-bound CA IV; mitochondrial CA VA and CA VB; secretory CA VI, CARP X and CARP XI; and transmembrane CA IX, CA XII and CA XIV (15).

Surprisingly, the first analyses of our study using the query bacterial β- and γ-CA sequences detected counterpart CA sequences in WZSP, and indeed, the MSA analysis approved that these sequences belong to the β- and γ-CA families. The BLAST search homology analyses of the identified β- and γ-CAs from WZSP displayed 100% identity to β- and γ-CA sequences from *Pseudomonas* spp. and *Acinetobacter* spp. In addition, genomic characterization of the detected β- and γ-CA sequences by the *Pseudomonas* Genome Database and Ensembl Bacteria database showed the presence of corresponding *β-* and *γ-CA* genes in the genomes of *Pseudomonas* spp. and *Acinetobacter* spp., with cytoplasmic subcellular localization of the encoded CAs.

Previous studies have revealed that both host gut-associated flora and environmental microbiome, such as airborne microbes as well as bacterial contamination of equipment and solutions used for DNA isolation, can represent potentially interfering substances and contamination sources of the shotgun metagenomic sequencing samples, leading to false-positive results (34–36). For similar reasons, it would be highly possible that the isolated DNA samples from WZSP for WGS project had been contaminated with bacterial members of the Pseudomonadales order including *Pseudomonas* spp. and *Acinetobacter* spp., resulting in the detection of β- and γ-CAs from these bacterial species in the Ensembl assembly (minipig_v1.0) of *S. scrofa*. In addition, further analysis with protein structure modeling of β- and γ-CA sequences from bacterial contaminants revealed that β-CA sequences from contaminants were similar to 5JJ8 crystal structure from *P. aeruginosa*, and γ-CA sequences from contaminants were similar to 3PMO crystal structure from *P. aeruginosa*, which both approve the membership of β- and γ-CA sequences of bacterial contaminants to Pseudomonadales order.

There are different pipelines for decontamination of genomic reads in DNA-Seq and RNA-Seq projects, such as hierarchical clustering algorithm (37), RapMap (38), DecontaMiner (39), Sequencing Quality Assessment Tool or SQUAT (40), map-guided scaffolding or MaGuS (41), and Kraken 2 (42), which can improve the quality of genomic samples. DNA-free reagents and kits are used to reduce the bacterial contamination in the sequencing projects (43). Internal controls of every step in the sequencing protocols can detect the trace fragments of foreign DNA or RNA to reduce the risk of bacterial contamination (44). Nevertheless, our results demonstrate that the sequences present in genomic databases do contain incorrect sequences due to microbial contamination, underlining the need for high-quality internal controls and biocuration.

## Conclusions

In addition to aforementioned methods for detection of bacterial contamination in the WGS projects of animals, the bioinformatics and computational approaches accompanied with bacterial-specific markers, such as CA sequences, can be employed to detect and reduce the risk of microbial contamination in the WGS projects through implementation of biocuration in databases. It is important to control the quality of short-size libraries, contigs and scaffolds as well as to perform internal checks of solutions, reagents and equipment during the shotgun genomic projects. This can be led to reducing the risk of annotation of false DNA and protein sequences in databases.
